# MicroRNA-155 Participates in the Expression of LSD1 and Proinflammatory Cytokines in Rheumatoid Synovial Cells

**DOI:** 10.1155/2020/4092762

**Published:** 2020-08-27

**Authors:** Ziliang Yu, Hao Liu, Jianbo Fan, Feihu Chen, Wei Liu

**Affiliations:** ^1^Department of Orthopaedics, Affiliated Hospital 2 of Nantong University, Nantong University, Nantong 226001, China; ^2^School of Clinical Medicine, Nanjing Medical University, Nanjing 211166, China

## Abstract

MicroRNA-155 (miRNA-155) is abundant in fibroblast-like synoviocytes (FLS) in rheumatoid arthritis (RA). Lysine-specific demethylase 1 (LSD1) has been found that it can ameliorate the severity of RA. Tumor necrosis factor-alpha, interleukin-1 beta, and interleukin-6 are key proinflammatory cytokines implicated in the pathogenesis of RA. In our study, we investigated whether miRNA-155 participates in the expression of LSD1 and proinflammatory cytokines in rheumatoid synovial cells. First of all, flow cytometry and cell counting kit-8 analysis were employed to explore the apoptosis and proliferation of FLS, respectively. Subsequently, reverse transcription-quantitative polymerase chain reaction (RT-qPCR) was applied to probe into the level of miRNA-155 in FLS when stimulated by miRNA-155 molecules. Moreover, RT-qPCR was used to explore the relative LSD1 miRNA expression in FLS when stimulated by miRNA-155 molecules, and Western blot and immunofluorescence assay were applied to probe into the expression level of LSD1. Finally, enzyme-linked immunosorbent assay was employed to analyze the secreting level of proinflammatory cytokines in FLS when stimulated by miRNA-155 molecules. RA-FLS showed a higher apoptosis rate than normal FLS. The cell proliferation of both HFLS and MH7A cells was promoted by miRNA-155 upregulation. Meanwhile, the expression of LSD1 and proinflammatory cytokines in the FLS of RA was also changed by miRNA-155 regulation. In conclusion, miRNA-155 participates in the expression of LSD1 and proinflammatory cytokines in rheumatoid synovial cells. These findings imply a potential function and interaction of miRNA-155 and LSD1.

## 1. Introduction

Rheumatoid arthritis (RA) is a kind of chronic autoimmune disorder featured by nonspecific inflammation of synovial membranes and joints. With the increased incidence rate and mortality percent, RA plagues approximately 1% of the world's population and brings enormous economic pressure and heavy social burden to the whole world [1, 2]. Earlier studies have shown that proinflammatory cytokines and inflammatory mediators are released in the pathological process of RA, which leads to chronic, symmetrical, and polysynovial arthritis. Moreover, these factors also cause recurrent and progressive joint pain and swelling, which eventually develop into joint damage, rigidity, and deformity, and even involve extraarticular organs [3, 4]. However, the mechanism of the pathological immune response of RA has not been elucidated.

Fibrosis exists in the pathological process of many diseases. There are large numbers of fibroblast-like synoviocytes (FLS) in human joints, which play considerable roles in the occurrence and persistence of RA. The synovial fluid produced by proliferative FLS increases the release of proinflammatory cytokines, which are crucial for both the joint destruction and the spread of inflammation in RA [5]. Tumor necrosis factor-alpha (TNF-*α*), interleukin-1 beta (IL-1*β*), and interleukin-6 (IL-6) are key proinflammatory cytokines implicated in the pathogenesis of RA [6, 7]. Targeted blocking of TNF-*α* and IL-6 is effective in the treatment of refractory RA patients [8, 9]. Chinese medicinal therapy is also a potential strategy to reduce the IL-1*β*-induced inflammation of RA [10].

Histone methylation is a dynamic process that influences the pathogenesis of RA. A considerable amount of studies have found that DNA methylation and histone modification may affect the progress of RA by coordinating the differentiation and function of immune cells [11, 12]. Therefore, regulation of histone methylation becomes a valid way to improve the RA treatment. Lysine-specific demethylase 1 (LSD1), a well-known lysine-specific demethylase, was initially found in 2004 [13]. Its function mainly depends on H3K4 (2 m) demethylase activity that brings an important physiological effect to many diseases [14]. Our previous research showed that LSD1-knockout mice effectively reduced RA development, especially decreased the joint injury and inflammatory response by abolishing LSD1 expression [15].

MicroRNAs (miRNAs), a class of small noncoding RNAs, are only 21-25 nucleotides. Many researchers focus on the implication of miRNAs in diseases. miRNA-155, as a member of the miRNAs family, regulates various diseases such as neoplastic diseases, cardiovascular disorders, and viral infection [16]. Moreover, miRNA-155 is crucial to the production of fibrosis; meanwhile, the dysfunction of miRNA-155 is closely related to the development of RA osteoclast differentiation [17–21]. miRNA-155^−/−^ mice showed reduced bone destruction and osteoclast production. Besides, miRNA-155 can regulate gene expression with the modification of histones in some regions [22], which implied a significant function of miRNA-155 to histone modification. Furthermore, it has been reported that KDM1A (LSD1) was a target of miRNA-155 in prostate cancer [23].

In view of the above, we aimed at proving whether miRNA-155 can also regulate the expression of LSD1 in RA and thus affect the secretion of proinflammatory cytokines. We used miRNA-155 mimics and inhibitor to stimulate HFLS and MH7A cells to explore the effects of miRNA-155 on cell proliferation, and the expression of LSD1 and proinflammatory cytokines.

## 2. Materials and Methods

### 2.1. Preparation of Cells

Human fibroblast-like synoviocytes (HFLS) and human rheumatoid FLS (MH7A) were purchased from BNCC (Beijing, China). Cells were cultured with Dulbecco's modified eagle medium (Gibco, Carlsbad, CA, USA) including 10% fetal bovine serum (Gibco, Carlsbad, CA, USA), penicillin, and streptomycin (Thermo Fisher Scientific, Waltham, MA, USA. Final concentration: streptomycin, 100 *μ*g/ml; penicillin, 100 U/ml). The cells were incubated in an incubator at 37°C and used at the 4^th^ generation.

### 2.2. Cell Apoptosis Detection

Apoptosis was measured using a flow cytometer (Guava Technologies, San Francisco, CA, USA) according to a previous study with some modification [24]. In brief, when the cell coverage rate reaches up to 75%, the drug induces apoptosis; the drug action time and its concentration have been explored in advance. The cell suspension was obtained from cells by D-hanks, 1× binding buffer washing, trypsin digestion, centrifugation, and resuspension. 5 *μ*l annexin V-FITC (Thermo Fisher Scientific, Waltham, MA, USA) was added to every 100 *μ*l cell suspension (1 × 10^5^ to 1 × 10^6^ cells). 5 *μ*l propidium iodide (PI, US Everbright® Inc., Suzhou, Jiangsu, China) staining (40 × PI dye solution diluted 10 times) was used for dyeing, and 1 × cell staining buffer was used to supplement the upper system to 300 *μ*l. All experiments in our study were performed independently at least three times for each point described.

### 2.3. Cell Transfection

miRNA-155 mimics and inhibitor (5 nmol/100 *μ*l), including their negative control (NC), were purchased (GenePharma Ltd., Shanghai, China). HFLS and MH7A cells in 6-well plates (2 × 10^5^ cells/well) were transfected using Lipofectamine 2000 reagent (Invitrogen, Carlsbad, CA, USA) according to the manufacturer's protocol.

### 2.4. Cell Proliferation Assay

Following transfection, cells were seeded into 96-well plates at a density of 2 × 10^3^ cells/well in a humidified atmosphere of 5% CO_2_ at 37°C for 24 hours. The viability of cells was determined using cell counting kit-8 (CCK-8, Dojindo, Kyushu, Japan) according to the manufacturer's protocol at 24, 48, 72, and 96 hours of culture. For detecting the proliferation ability, the CCK-8 reagents were added in each well (10 *μ*l/well). After incubating at 37°C with 5% CO_2_ for 1.5 hours, the optical density was measured at a wavelength of 450 nm. The proliferation curve was plotted with GraphPad Prism 8.0.

### 2.5. Reverse Transcription-Quantitative Polymerase Chain Reaction (RT-qPCR)

Total RNA extraction and RT-PCR were performed according to a previous study with some modification [25]. Total RNA was extracted from the passage 4 cells according to the instructions of TRIzol (Sigma, Shanghai, China). Briefly, cells were collected by centrifugation at 2000 rpm for 5 minutes and then mixed with 1 ml of TRIzol at room temperature for 5 minutes. Subsequently, the lysates were shaken for 10 seconds and stood at room temperature for 10 minutes. After centrifuging, the lysates at 12800 rpm at 4°C for 10 minutes, the supernatant was mixed with isochoric precooling isopropanol for 10 minutes and centrifuged at 12800 rpm and 4°C for another 10 minutes. 75% ethanol was used to wash the precipitate twice. When the precipitate was dry, it was dissolved in 30 *μ*l RNase-free water (Thermo Fisher Scientific, Waltham, MA, USA) and quantified by a NanoDrop 100 spectrophotometer (ALLSHENG, Hangzhou, Zhejiang, China).

cDNA was synthesized according to the instructions of HiScript® Q RT SuperMix for qPCR (Vazyme, Nanjing, Jiangsu, China). Briefly, 4 × gDNA wiper Mix and 1 *μ*g of total RNA were added to the PCR tube, then RNase-free H_2_O was complemented to 8 *μ*l. After mixing, centrifugation was performed, and bathing at 42°C for 2 minutes. Subsequently, 4 *μ*l 5 × qRT SuperMix II was added to the PCR tube. The following thermocycling conditions were applied for the cDNA synthesis: 55°C for 15 minutes and 85°C for 2 minutes. The obtained cDNA was stored at -80°C for future use.

SYBR Green mastermixes and a ViiA™ 7 real-time PCR system with 384-well block (Thermo Fisher Scientific, Waltham, MA, USA) were utilized to perform the PCR analysis. The information of forward and reverse primers is listed in Table 1. The following thermocycling conditions were applied for the PCR: Initial denaturation for 5 minutes at 95°C; 40 cycles of 95°C for 10 seconds, 60°C for 30 seconds; 1 cycle of dissociation for 95°C for 15 seconds, 60°C for 60 seconds and 95°C for 15 seconds. The expression level was normalized using glyceraldehyde-3-phosphate dehydrogenase (GAPDH) small nuclear RNA and quantified using the 2^-*ΔΔ*Cq^.

### 2.6. Western Blot (WB)

Protein extraction and WB were performed as previously described, with some modification [26]. Protein was extracted from the passage 4 cells when the cell coverage rate reaches up to 90% according to the instructions of SDS lysis buffer (Beyotime, Shanghai, China). The cells taken from the incubator were washed twice with phosphate buffer saline (Beyotime, Shanghai, China) after discarding the culture solution, then added to the cell lysate and placed on ice at 4°C. Blew with the tip of the pipette until the cells are fully lysed, transferred the lysed samples into the centrifuge tube, and continued to lyse on the ice for 10 minutes. After cracking, the supernatant was transferred to a new centrifuge tube and centrifuged at 4°C, 12000 rpm for 10 minutes, adding loading buffer at 100°C, 20 minutes; after water bath, the centrifuge tube at 4°C, 12000 rpm, centrifuged for 1 minute, and stored at -20°C for standby. Subsequently, a total of 12 *μ*g of protein was loaded into SDS-PAGE according to the instructions of the SDS-PAGE gel preparation kit (Beyotime, Shanghai, China), and transferred to polyvinylidene fluoride membranes (Thermo Fisher Scientific, Waltham, MA, USA). The membranes were blocked with Tris-buffered saline Tween-20 (TBST, Beyotime, Shanghai, China) containing 5% skimmed milk (FUJIFILM Wako Pure Chemical Corporation, Osaka, Japan) dissolved in for 1 hour at room temperature. Subsequently, the membranes were rinsed with TBST three times and incubated with primary antibody dissolved in antibody diluted by blocking solution at room temperature for 2 hours. Membranes were then incubated with the corresponding secondary antibodies (Rabbit. no. Ab129195; 1 : 10,000, Abcam, Shanghai, China) at room temperature for 1 hour. Protein bands were visualized using the Image J (1.51j8 version, Bethesda, MD, USA). The expression level of the targeted protein was normalized to that of GAPDH.

### 2.7. Immunofluorescence Assay (IFA)

To investigate the effect of miRNA-155 on the expression and distribution of LSD1 in cells, the fixed cell slides were blocked with 3% H_2_O_2_ for 5 minutes, permeabilized with 0.5% Triton X-100 (Solarbio, Beijing, China) for 30 minutes, and then blocked with 5% rabbit serum albumin for 15 minutes. Subsequently, the cells were incubated with mouse-derived antibody diluted in 0.1% Triton at 37°C for 1 hour, followed by a further incubation at 37°C for 30 minutes with a rabbit secondary antibody to Mouse IgG (Anti-KDM1A/LSD1, ab129195, Abcam, Shanghai, China) at 1 *μ*g/ml (shown in green). Nuclear DNA was labeled in blue with DAPI. The slides were photographed with blue light, and the expression of antibodies in tissues was determined according to the distribution of nuclei under blue light.

### 2.8. Enzyme-Linked Immunosorbent Assay (ELISA)

To determine the effect of miRNA-155 and LSD1 on the secretion of proimmunology cytokines by FLS, HFLS and MH7A cells were transfected with miRNA-155 mimics and inhibitor. A total of 48 hours following transfection, the concentration of TNF-*α*, IL-1*β*, and IL-6 in the cell culture supernatant (obtained by centrifugation at 13500 rpm for 5 minutes at room temperature) was performed using ELISA kits (mIbio, Shanghai, China) according to the instructions.

### 2.9. Statistics Analysis

All data are presented as mean ± standard deviation. The comparison between every two groups was evaluated using an unpaired two-tailed *t*-test. It was considered to be statistically significant that the *P* value was less than 0.05. All statistical analyses were performed using GraphPad Prism 8.0 for Windows (San Diego, CA, USA).

## 3. Results

### 3.1. RA Induces the Cell Apoptosis of FLS

**Figure 1:**
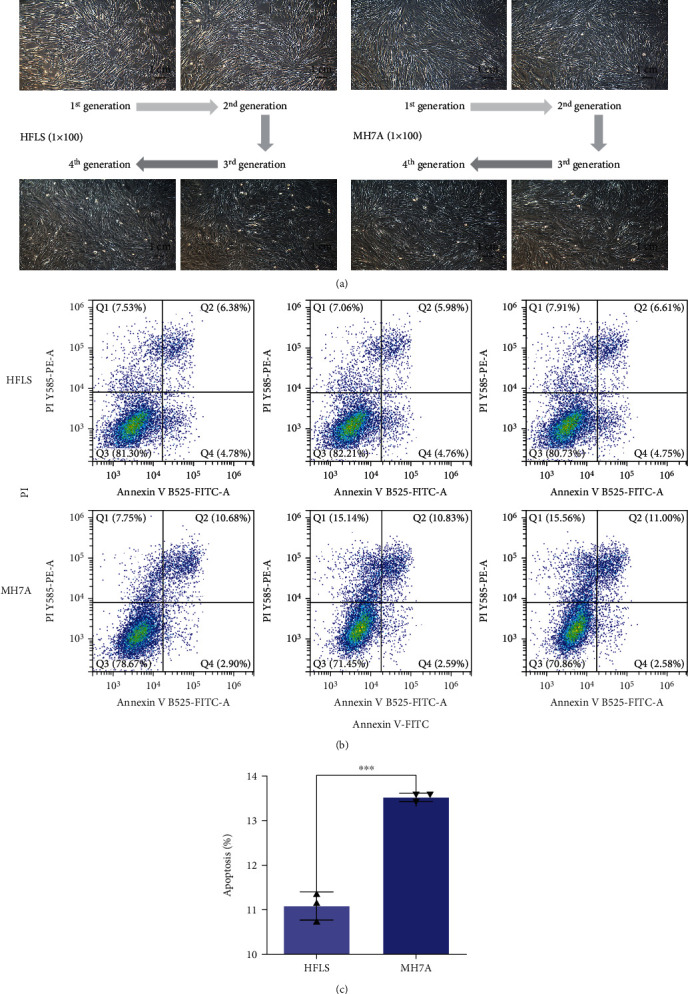
The growth and apoptosis of normal FLS and RA-FLS. (a) HFLS and MH7A cells were cultured and observed until the 4^th^ generation. Scale bar, 1 cm. (b, c) The apoptosis of HFLS and MH7A cells was evaluated by flow cytometry. ^∗^*P* < 0.05.

To investigate whether RA causes the cell apoptosis, we set out to identify the cell apoptosis ratio between normal FLS and rheumatoid FLS. HFLS and MH7A cells were cultured until the 4^th^ generation (Figure 1(a)) and analyzed by flow cytometry (Figure 1(b)). The histogram analysis indicated that the apoptotic ratio of MH7A cells is significantly higher than that of HFLS cells (Figure 1(c)). The results showed that RA can induce cell apoptosis easily.

### 3.2. Upregulation of miRNA-155 Promotes the Proliferation of HFLS and MH7A Cells

**Figure 2:**
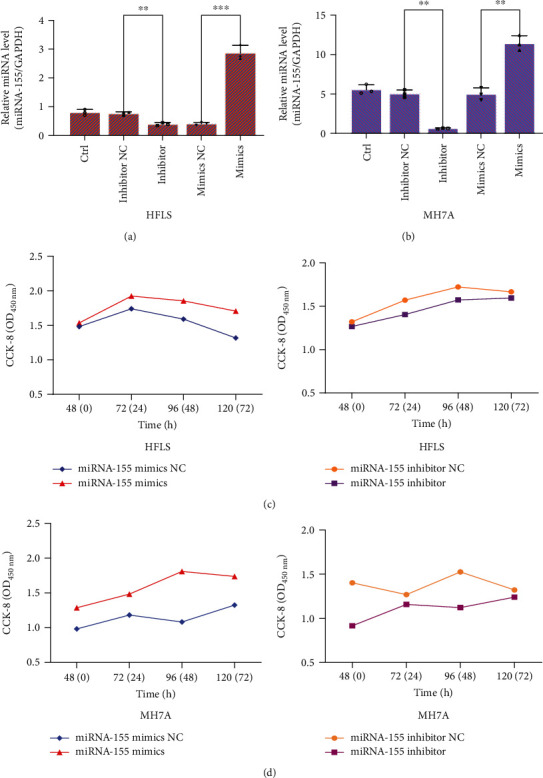
The effects of miRNA-155 on cell viability of normal FLS and RA-FLS. (a, b) Both HFLS and MH7A cells (2 × 10^5^ cells/well) were transfected with 5 *μ*l miRNA-155 mimics and inhibitor for 48 hours. Total RNA was extracted to detect the expression of miRNA-155 by RT-qPCR. Results were normalized by GAPDH miRNA. ^∗^*P* < 0.05. (c) HFLS cells were transfected with miRNA-155 mimics and inhibitor for 48 hours, followed by the assessment of cell viability by CCK-8 assay. ^∗^*P* < 0.05. (d) MH7A cells were transfected with miRNA-155 mimics and inhibitor for 48 hours, followed by the assessment of cell viability by CCK-8 assay. ^∗^*P* < 0.05.

Unlike the cancer cell proliferation [24, 27], the mechanism of RA cell proliferation remained unknown. We next used miRNA-155 to identify its regulation on the proliferation of HFLS and MH7A cells. We firstly checked the expression of miRNA-155 in these two groups of cells and after being interfered by miRNA-155 mimics and inhibitor. RT-qPCR results indicated that the miRNA-155 level of MH7A cells was significantly higher than that of HFLS cells, and it can be upregulated or downregulated individually after miRNA-155 mimics or inhibitor treatment in HFLS and MH7A cells (Figures 2(a) and 2(b)). Following transfection with miRNA-155 mimics and inhibitor, CCK-8 assay was used to determine the proliferation of HFLS and MH7A cells (Figures 2(c) and 2(d)). The results displayed that miRNA-155 mimics can promote the proliferation of both HFLS and MH7A cells. Consistently, miRNA-155 inhibitor also inhibits the proliferation of both HFLS and MH7A cells.

### 3.3. Upregulation of miRNA-155 Decreases the Expression of LSD1 in RA

**Figure 3:**
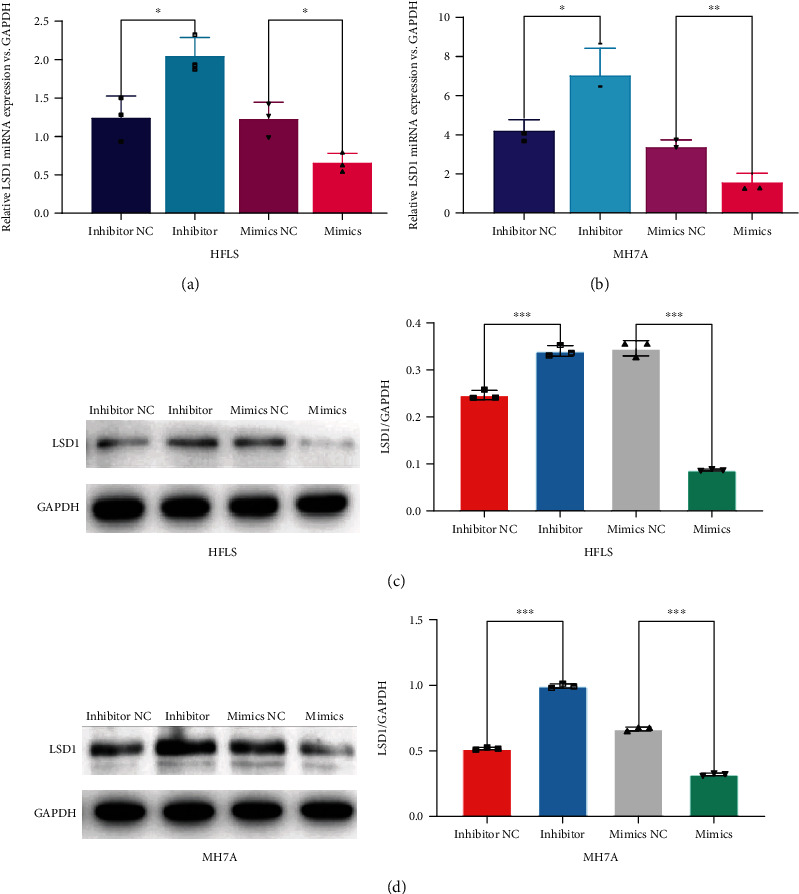
The regulation of miRNA-155 on LSD1 expression in normal FLS and RA-FLS. (a, b) Both HFLS and MH7A cells were transfected with miRNA-155 mimics and inhibitor for 48 hours. Total RNA was extracted to detect the expression of LSD1 miRNA by RT-qPCR. Results were normalized by GAPDH miRNA. ^∗^*P* < 0.05. (c, d) The protein levels of LSD1 in both HFLS and MH7A cells were detected by Western blot. Results were normalized by GAPDH protein. ^∗^*P* < 0.05.

LSD1 has been known as a target of miRNA-155. To further clarify the effect of miRNA-155 on LSD1, we detected the expression level of the relative LSD1 miRNA in HFLS and MH7A cells after miRNA-155 mimics and inhibitor interference. We treated cells with miRNA-155 mimics and inhibitor to identify the relationship between LSD1 and miRNA-155. RT-qPCR results showed that the miRNA level of LSD1 in the inhibitor group increased. Correspondingly, the miRNA level of LSD1 decreased in the mimics group (Figures 3(a) and 3(b)). Moreover, LSD1 protein expression was also regulated by miRNA-155 mimics and inhibitor, which was consistent with the RT-qPCR data (Figures 3(c) and 3(d)).

**Figure 4:**
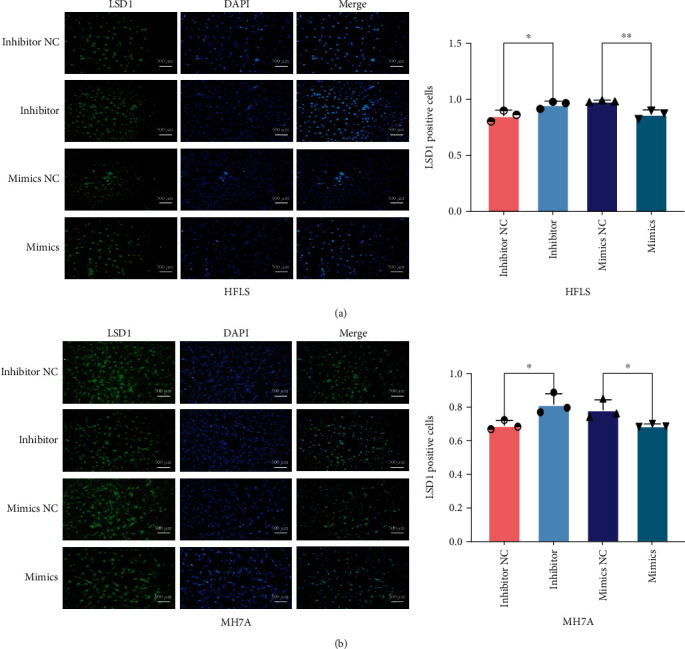
The cellular distribution of LSD1 in normal FLS and RA-FLS when transfected with miRNA-155 molecules. (a) Representative imaging of LSD1 distribution in both HFLS and MH7A cells. Immunofluorescence staining used LSD1 antibody (green) and DAPI (blue). Scale bar, 500 *μ*m. (b) Semiquantitative analysis of (a). ^∗^*P* < 0.05.

In order to further verify the effect of miRNA-155 on the expression of LSD1, we used IFA to observe whether the distribution of LSD1 changed in cells stimulated by treating miRNA-155 mimics and inhibitor. We observed that the distribution of LSD1 was in the nucleus, and its expression in both two cells was stimulated significantly by miRNA-155 inhibitor treatment. On the contrary, LSD1 expression decreased in the miRNA-155 mimics group (Figures 4(a) and 4(b)). These results indicated that miRNA-155 can target and negatively regulate LSD1 in both HFLS and MH7A cells.

### 3.4. Upregulation of miRNA-155 Decreases the Secretion of Proinflammatory Cytokines in RA

**Figure 5:**
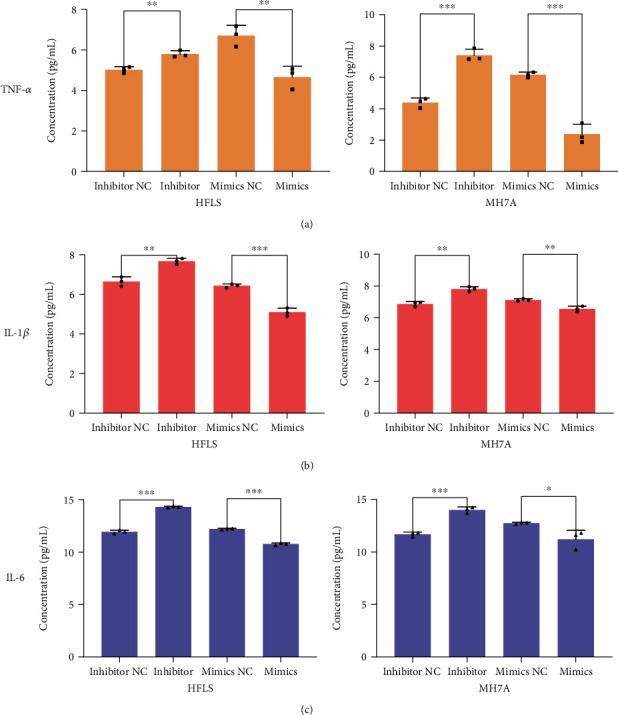
The effects of miRNA-155 on the production of proinflammatory cytokines in normal FLS and RA-FLS. (a) The levels of TNF-*α* in the supernatant of both HFLS and MH7A cells were measured by ELISA assay. ^∗^*P* < 0.05. (b) The levels of IL-1*β* in the supernatant of both HFLS and MH7A cells were measured by ELISA assay. ^∗^*P* < 0.05. (c) The levels of IL-6 in the supernatant of both HFLS and MH7A cells were measured by ELISA assay. ^∗^*P* < 0.05.

It is known that multiple proinflammatory cytokines were secreted during RA, including TNF-*α*, IL-1*β*, and IL-6 [6, 7]. Here, we attempt to find out whether miRNA-155 participates in regulating the secretion of TNF-*α*, IL-1*β*, and IL-6 in HFLS and MH7A cells. The secretion of TNF-*α*, IL-1*β*, and IL-6 in cell supernatant was examined by ELISA, respectively. Data analysis showed that these factors were all markedly raised in miRNA-155 inhibitor-treated cells compared with the miRNA-155 inhibitor NC group. Similarly, their secretion also decreased by the treatment of miRNA-155 mimics (Figure 5). Thus, these results revealed that miRNA-155 is associated with the expression of some proinflammatory cytokines in RA.

## 4. Discussion

RA is characterized by the improved proliferation of FLS and aggregation of proinflammatory cytokines in the joints. Previous studies have shown that RA is mainly due to the improved proliferation, migration, and invasion capacity of FLS [28]. Some evidence showed that miRNAs play huge roles in immune system diseases [29–32]. In addition, some miRNAs modulated by drug treatment also have important effects on osteogenic differentiation. For example, miR-21-5p can affect MC3T3-E1 cell proliferation, apoptosis, osteogenic differentiation, and matrix mineralization of osteoblasts by Icariin [33], which is known as an antitumor drug [25, 26]. However, the specific role of miRNA-155 in RA has not been elucidated.

In our study, we firstly cultured the obtained HFLS and MH7A cells and detected their apoptosis percent at the 4^th^ generation, the results showed that the apoptosis percent of MH7A cells was significantly higher than that of HFLS cells. It may be due to that MH7A cells contain more inflammation factors and possess stronger migration and invasion abilities than HFLS cells.

Subsequently, we used miRNA-155 mimics and miRNA-155 inhibitor to stimulate HFLS and MH7A cells, we randomly divided the samples into two groups; one group was used to detect the expression of miRNA-155 in cells by RT-qPCR, and the other group was used to detect the cell proliferation by CCK-8 analysis. The results suggest that miRNA-155 mimics can promote the expression of miRNA-155 and the proliferation of FLS cells (not only normal FLS but also RA-FLS). During the occurrence of some autoimmune diseases, miRNA-155 has been proved to promote cell proliferation [34, 35]. We also found the same phenomenon in RA, which may further explain the function of miRNA-155 on cell proliferation in autoimmune diseases. In addition, some studies have shown that miRNA-155 abounds in B cells, monocytes, and macrophages from synovial tissues of RA patients [36, 37]. On this basis, our results showed a similar conclusion in inflammatory cells but more clearly elucidated a direct function of miRNA-155 on inflammatory cell proliferation.

In our study, RT-qPCR and WB results showed that miRNA-155 mimics and inhibitor treatment regulated the expression of LSD1, especially in MH7A cells. These results implied that miRNA-155 can affect cell proliferation through LSD1, and its effect was obvious in the inflammatory cells. Although LSD1 has been reported to promote the cell proliferation in some tumor diseases [38, 39], its function in autoimmune diseases, especially in RA, still needs to be studied. Our results provided new evidence to elucidate that miRNA-155 related cell proliferation may through LSD1 regulation. Therefore, the molecular mechanism of LSD1 in RA will be our future research direction to explore the possible targeted therapy.

In order to explore whether miRNA-155 can affect joint inflammation by regulating LSD1, we used miRNA-155 mimics and inhibitor to interfere with HFLS and MH7A cells. The results showed that miRNA-155 mimics could inhibit the expression of LSD1 and proinflammatory cytokines in both HFLS and MH7A cells. On the contrary, miRNA-155 inhibitor could promote the expression of LSD1 and proinflammatory cytokines in both HFLS and MH7A cells. However, several studies found that miRNA-155^−/−^ mice showed reduced bone destruction and osteoclast production [22]. Moreover, overexpression of miRNA-155 in monocytes leads to an increase of TNF*α*, IL-1*β*, and IL-6 [40]. Our previous study showed that the joint injury and inflammatory response of RA model mice were improved to a certain extent after the LSD1 gene knockdown [15]. The complexity of RA and the different cell types may be the main reasons for the different conclusions. However, the interaction of miRNA-155 and LSD1, which closely related to the inflammation, could identify the potential roles in FLS cells. It will guide us to further investigate the function of LSD1 during inflammation, and to confirm that miRNA-155 can regulate proteins to affect the expression of proinflammatory cytokines [29].

Although our research cannot cover the whole process of RA, we try to explain the development of inflammation. The internal environment of the body is quite complicated. A certain gene, protein, or factor alone cannot determine the occurrence and outcome of the disease. Due to the change of culture conditions and the adaptability of the cell itself, the in vitro environment will affect cell proliferation, apoptosis, invasion, secretion, etc. We hope that our research will help to provide more evidence and new strategies for drugs and the fundamental prevention and treatment of RA.

## Figures and Tables

**Table 1:** Primer sequences and their amplified bands' size range. Sequences are listed 5′→3′.

Gene	Primer (forward)	Primer (reverse)	Length (bp)
GAPDH	CTCGCTTCGGCAGCACA	AACGCTTCACGAATTTGCGT	96
miRNA-155	GCGCCTCCTACATATTAGCA	GTGCAGGGTCCGAGGT	77

## Data Availability

The data used to support the findings of this study are available from the corresponding author upon request.
